# Improved tribological performance of silica glass *via* surface texturing by chemical etching

**DOI:** 10.1039/d5ra06230f

**Published:** 2025-11-10

**Authors:** Sung-Jun Lee, Chang-Lae Kim

**Affiliations:** a Department of Mechanical Engineering, Chosun University Gwangju 61452 Republic of Korea kimcl@chosun.ac.kr

## Abstract

This research examined the impact of chemical etching on the friction and wear resistance of glass surfaces. Glass samples were etched using deionized water and ammonium bifluoride solution for 1–60 minutes. The surface morphology, roughness, and wettability were analyzed, and reciprocating sliding tests were performed to evaluate the tribological performance of the etched surfaces compared to bare glass. Finite element analysis was used to examine the stress distribution during indentation. The results showed that chemical etching created micro- and nanoscale surface features that significantly reduced the friction coefficients and improved the wear resistance compared to bare glass, with 10-minute etching showing optimal performance. The enhanced tribological behavior resulted from surface texturing, which dispersed the contact stresses, promoted stable lubricating film formation, and reduced direct asperity contact. This demonstrates the potential of chemical etching as an effective surface modification technique for improving the friction performance of glass in industrial applications.

## Abstract 2

This research examined the impact of chemical etching on the friction and wear resistance of glass surfaces.

## Graphical abstract

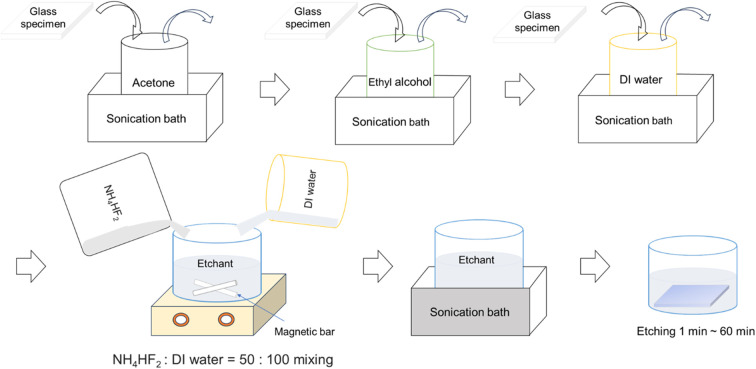

## 1. Introduction

Surface engineering has emerged as a crucial field in materials science, focusing on modifying and optimizing the surface properties to improve the performance and durability of materials across various applications.^1–3^ Among the numerous surface characteristics, surface roughness is a critical parameter that significantly influences the friction and wear characteristics of contacting materials in a wide range of applications such as mechanical components, tribological systems, micro/nano devices, and advanced material processing.^4,5^ The topography of the contacting surfaces plays a pivotal role in determining the actual contact area, stress distribution, and governing mechanisms of friction and wear.^6,7^ By controlling the surface roughness, it is possible to tailor the frictional performance, improve the durability and reliability of the contacting components, minimize energy losses, reduce maintenance costs, and extend the operational life of these systems.

Numerous experimental and theoretical studies have been conducted to elucidate the complex relationship between surface roughness and friction/wear behavior.^7–9^ However, owing to the complexity of the underlying mechanisms and the interplay of various factors, such as material properties, surface chemistry, environmental conditions, and loading conditions, understanding this relationship remains challenging. The influence of surface roughness on friction and wear is governed by several mechanisms including plastic deformation, plowing, adhesion, wear, and third-body interactions.^10,11^

In this study, we aimed to investigate the effect of surface roughness on friction and wear behavior using a synergistic approach that combines experimental and computational methods. Glass substrates were chosen as the model material because of their widespread application in various industries, such as optics, displays, and microfluidics, as well as their well-established etching protocols for controlling surface modifications.^12–14^

Glass is a versatile material with excellent optical, thermal, and chemical properties, widely used in various industrial sectors, including automotive, aerospace, electronics, and biomedical applications.^15–18^ Although glass is highly valued for its transparency in many applications, there are specific areas where the surface roughness of glass does not significantly affect its functionality. In such cases, the tribological performance of glass, particularly its friction and wear characteristics, is a crucial factor in determining its suitability and durability for intended applications.

In the automotive industry, glass is used in various components, such as windshields, side windows, and rearview mirrors. Although the surface roughness of these components can impact optical clarity and visibility, there are other glass components where surface roughness does not play a significant role. Engine components such as cylinder liners and piston rings can benefit from glass-based materials with improved friction properties.^19^ The ability to reduce friction and wear in these components can lead to improved engine efficiency, reduced fuel consumption, and an extended component life.

Similarly, in the aerospace industry, glass-based materials are used in various applications in which surface roughness does not significantly affect functionality. In satellite communication systems, glass is used for the fabrication of antenna radomes.^20^ The primary requirement of these radomes is to protect the antenna from environmental conditions while minimizing signal attenuation. In this case, the tribological performance of the glass material is a critical factor because it determines the durability and reliability of the radome under harsh operating conditions.

In the biomedical field, glass-based materials are used in various implantable devices such as bone scaffolds and dental implants.^21^ Although surface roughness can influence cell adhesion and tissue integration in some applications, there are cases where the frictional characteristics of the glass material are more crucial. For instance, in load-bearing implants, such as artificial hip and knee joints, the ability to reduce friction and wear at the articulating surfaces is of utmost importance for the long-term success and durability of the implant.^22^ Glass-based materials with improved tribological properties can contribute to the development of high-performance long-lasting implantable devices.

Surface engineering techniques have emerged as promising approaches for improving the tribological performance of glass materials.^23^ Among these techniques, chemical etching has gained attention as a simple and effective method for modifying the surface morphology and roughness of glass.^24^ By selectively removing the material from the glass surface using corrosive solutions, chemical etching can create micro- and nanoscale surface features that significantly influence the tribological behavior of the material.^25^

In this study, we controlled the surface roughness of glass through chemical etching and investigated the resulting changes in its frictional properties. The glass samples were treated for various etching times using deionized water and ammonium bifluoride solutions, and the surface morphology and roughness were analyzed using optical microscopy, scanning electron microscopy, and surface profiling techniques. In addition, the wettability of the surfaces was evaluated using contact-angle measurements. To assess the frictional performance of the etched glass surfaces, reciprocating sliding tests were conducted using a ball-on-plate configuration, and the friction and wear behaviors were investigated through friction coefficient monitoring and wear track analysis. The correlation between the surface roughness and frictional characteristics was elucidated through experimental results and finite element analysis simulations, and the underlying mechanisms are discussed. The findings of this study contribute to the fundamental understanding of surface roughness effects and can potentially impact the design and optimization of tribological systems in various industrial sectors, including mechanical components, micro/nano devices, and advanced material applications. By tuning the surface roughness, it is possible to improve the frictional performance, reduce energy losses, minimize wear, and extend the operational life of systems, ultimately improving efficiency, reliability, and sustainability.

While long-term tribological performance over extended sliding distances on the order of kilometers remains a critical consideration for many industrial applications, the improved initial-stage tribological behavior demonstrated in this study is particularly relevant for specific applications. These include precision positioning systems and micro-mechanical devices where operation involves limited sliding distances, start–stop mechanisms in automotive applications where initial friction reduction is crucial, medical devices with intermittent motion patterns, MEMS/NEMS devices where cumulative sliding distances remain relatively low, and optical alignment systems requiring smooth initial motion characteristics. This study focuses on understanding the fundamental mechanisms of friction reduction during the initial sliding phase, which provides a foundation for developing longer-lasting surface treatments and applications where short-distance tribological performance is critical.

## 2. Materials and methods

### 2.1 Materials

To evaluate the surface, structural, chemical, frictional, and wear properties of the glass specimens as a function of etching time, the following materials were used. Soda-lime-silica glass (Paul MarienfeldGmbH&Co., KG, Lauda-Königshofen, Germany), acetone (99.5%, Duksan Pure Chemicals Co. Ltd, Ansan, Republic of Korea), ethanol (99.5%, Duksan Pure Chemicals Co. Ltd, Ansan, Republic of Korea), deionized water (ultra pure, Duksan Pure Chemicals Co. Ltd, Ansan, Republic of Korea), and ammonium bifluoride (NH_4_HF_2_; 97%, Daejung Chemicals & Metals Co. Ltd, Siheung, Korea).

### 2.2 Specimen preparation

The etching solution was prepared by dissolving 50 g of ammonium bifluoride in 100 g of deionized water, resulting in a 33.3 wt% NH_4_HF_2_ solution. The mixture was magnetically stirred at 400 rpm for 3 h to ensure homogeneity. Additionally, the etching solution was ultrasonicated for 1 h to achieve uniform dispersion.

**Fig. 1:**
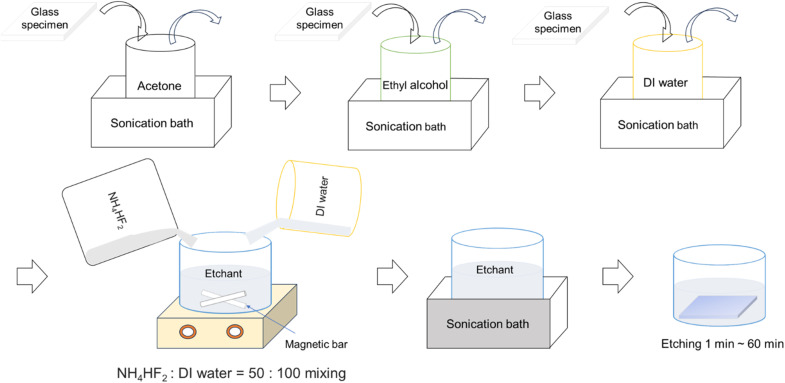
Schematic representation of specimen fabrication process for etched glass samples.

Glass specimens were fabricated using various processes, as shown in Fig. 1, to ensure consistency and reliability. First, the glass specimens were ultrasonically cleaned in acetone, ethanol, and deionized water for 10 minutes each. Subsequently, the cleaned glass specimens were immersed in the pre-prepared etching solution for durations ranging from 1 to 60 min. After etching, the specimens were ultrasonically cleaned in acetone, ethanol, and deionized water for 20 min each. Finally, the etched glass samples were dried in a heating chamber at 60 °C for 2 h to remove any residual moisture.

The etching mechanism of silica glass with NH_4_HF_2_ solution proceeds through the following reaction:^26,27^

1 SiO_2_ + 4HF → SiF_4_ + 2H_2_O

The dissolution reaction is governed by the adsorption of reactive species HF and HF_2_^−^ and the catalytic action of H^+^ ions, resulting in the breakage of siloxane bonds in the silicate network. NH_4_HF_2_ dissolves in water to release HF ions in a controlled manner, which react with silica present in glass.

The unetched specimen is referred to as bare, while the etched specimens are denoted as 1, 5, 10, 30, and 60 min, corresponding to etching times of 1, 5, 10, 30, and 60 min, respectively.

### 2.3 Experiments

Various techniques were employed to analyze the surface characteristics and frictional behavior of the etched glass specimens. Optical microscopy (DM750, Leica, Wetzlar, Germany) were used to observe the surface morphology before and after cleaning to analyze the influence of etching time on the surface topography.

X-ray diffraction (XRD; EMPyrean, PANalytical, Malvern, UK) analysis was performed using Cu Kα radiation (*λ* = 1.5406 Å) at 40 kV and 30 mA, with a scan rate of 0.02° s^−1^ in the 2*θ* range of 5–90° to investigate the crystalline structures of the bare and etched glass samples. Fourier transform infrared (FTIR) spectroscopy (Spectrum 3, PerkinElmer, Massachusetts, USA) was conducted in the wavenumber range of 650–4000 cm^−1^ to identify the functional groups present on the specimen surfaces.

The wettability of the glass surfaces was evaluated by measuring the contact angle of the water droplet using the drop method. A 5 μL droplet of deionized water was placed on the specimen surface, and the contact angle was measured using a microscopic camera. Surface roughness was assessed using a contact-type 2D profiler (SV-2100M4, Mitutoyo Korea Corporation, Gunpo, Korea) equipped with a diamond stylus tip, operating at a load of 0.75 mN, a scanning speed of 0.1 mm s^−1^, and a distance of 1 mm. To ensure reliability, the contact angle and surface roughness measurements were repeated at least five times and the average values were calculated.

Friction tests were conducted using a reciprocating tribometer (RFW 160, NEOPLUS, Co., Ltd, Daejeon, Korea) with a 304 stainless-steel ball (diameter: 1 mm) as the counter tip. Two sets of tests were performed. In the first set, a normal load of 20 mN was applied, and the counter tip was reciprocated for 2000 cycles at a speed of 4 mm s^−1^ over a sliding distance of 2 mm. In the second set, normal loads of 20, 30, and 50 mN were applied, and the counter tip was reciprocated for 5000 cycles with the same sliding distance and speed. For each condition, the friction coefficient was measured at least three times, and the average values were used for analysis. The wear track analysis was performed using both scanning electron microscopy (SEM; SU-8100, Hitachi, Tokyo, Japan) and contact profilometry. Surface roughness within wear tracks was measured to the sliding direction at five positions along each track using the same profiler settings as initial surface characterization.

Finite element analysis (FEA) was performed to simulate the contact behavior during indentation. In the FEA simulation, the etched surface was modeled by incorporating the measured surface roughness profile. Based on the RufGen plug-in methodology, we applied a sinusoidal surface texture with parameters derived from 10-minute etched sample.^28^ The bare glass was modeled as an ideally smooth surface without any texture. The elastic modulus and Poisson's ratio of the 304 stainless steel ball were set to 210 GPa and 0.35, respectively, whereas those of the glass were set to 64 GPa and 0.2, respectively.^29,30^ The FEA model was constructed using a static, general analysis step. A reference point was designated at the upper surface of the counter tip and coupled to control the loading condition. The contact between the counter tip and glass substrate was defined using surface-to-surface contact interaction. For mesh generation, both the substrate and counter tip were meshed with an approximate global size of 0.01 mm. The substrate employed quad-dominated element shapes for improved accuracy in stress distribution analysis, while the counter tip used triangular (Tri) element shapes to better conform to its spherical geometry. Indentation simulations were performed under normal loads of 20, 30, and 50 mN, with the load applied through the reference point in the vertical direction.

## 3. Results and discussion

**Fig. 2:**
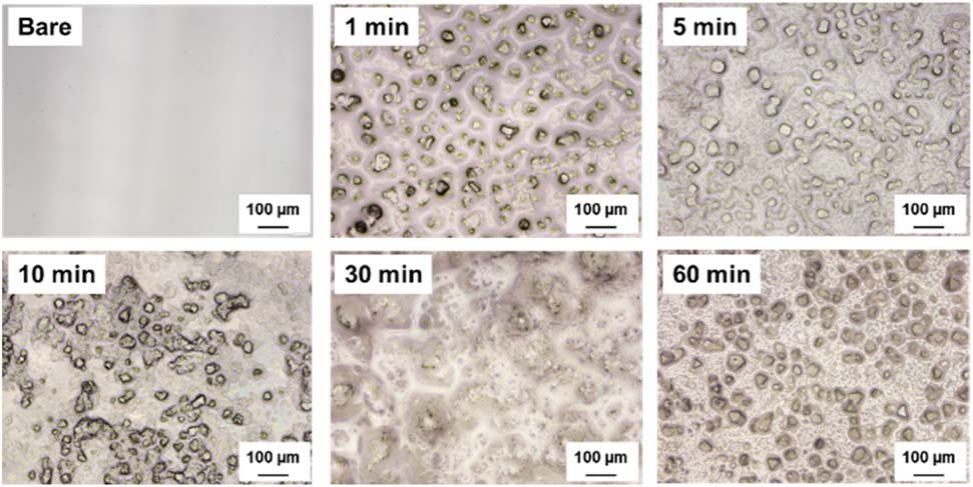
Optical microscopy images of the glass surface morphologies before cleaning.

Fig. 2 shows the surface morphologies of the glass samples before cleaning, as observed using an optical microscope. The bare glass sample exhibited a relatively smooth surface with minimal features, which served as a reference for comparing the effects of etching. As the etching time increased from 1 min to 60 min, the surface morphology underwent significant changes. The 1-minute etched sample showed the initial stages of surface texturing, with subtle surface features appearing and a slight increase in roughness. These features become more pronounced in the 5-minute etched sample, indicating a gradual increase in surface roughness. The 10-minute and 30-minute etched samples reveal further evolution of the surface texture, with the formation of distinct micro- and nanoscale features. These features were distributed across the surface, creating a more complex and heterogeneous topography. The 60-minute etched sample exhibited the most pronounced surface texturing, with a high density of well-defined micro- and nanoscale structures. The surface morphology before cleaning could be attributed to the chemical etching process. During etching, the interaction between the glass surface and etching solution results in selective material removal, leading to the formation of surface irregularities and features.^31^ The extent and characteristics of these features vary with etching time, with longer durations allowing for more extensive material removal and the realization of more complex surface textures. The etching process is governed by the chemical reactions occurring at the glass-etchant interface, where the etchant selectively dissolves specific components of the glass network. This selective dissolution contributes to the creation of micro- and nanoscale voids, pits, and protrusions on the surface, thereby increasing surface roughness.

**Fig. 3:**
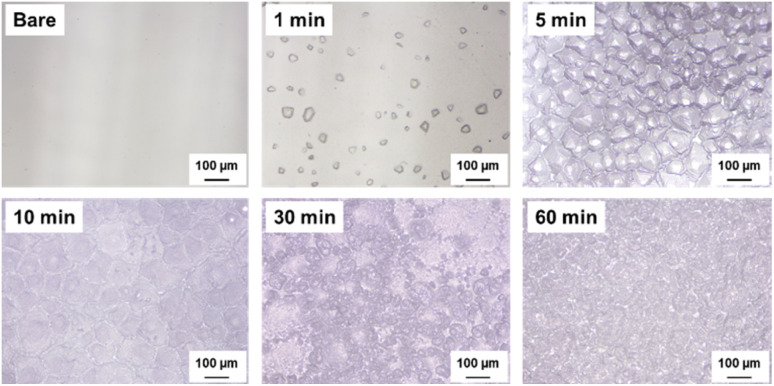
Optical microscopy images of the glass surface morphologies after cleaning.

Fig. 3 presents the surface morphology of the glass samples after the etching and subsequent cleaning processes. Cleaning plays a crucial role in removing any residual etching solution, debris, or contaminants from the surface, thereby ensuring a clean and well-defined surface texture. The bare glass sample maintained a smooth surface after cleaning, with no significant changes observed. However, the etched samples exhibited noticeable changes in surface morphology after cleaning. The 1-minute etched sample revealed a cleaner and more distinct surface texture compared to its pre-cleaning state, with subtle surface features becoming more visible. The 5-minute, 10-minute, and 30-minute etched samples show a gradual improvement in the surface roughness and clarity of the micro- and nanoscale features. The cleaning process effectively removed any imperceptible residues, allowing the true surface texture to be observed. The 60-minute etched sample exhibited the most striking change after cleaning, with highly complex and well-defined surface features fully exposed. The cleaning step following the etching treatment ensured the removal of any remaining etching solution from the surface, preventing further uncontrolled etching and ensuring a stable surface morphology. The cleaning stage can remove any debris or particles generated during the etching process, which may interfere with surface characteristics and frictional performance. Moreover, cleaning helps reveal the actual surface texture created by the etching process, enabling a more accurate assessment of the surface roughness and morphology.

**Fig. 4:**
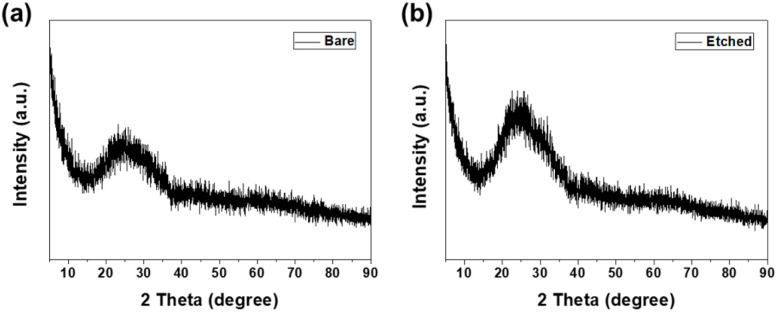
X-ray diffraction patterns of (a) bare and (b) etched glass samples.

Fig. 4 shows the XRD patterns of the bare and etched glass samples, confirming the amorphous nature of the glass substrates. Both samples exhibited a broad peak centered at approximately 25°. The broad peak at approximately 25° is characteristic of amorphous materials and indicates the absence of long-range crystalline order.^32^ The absence of sharp peaks in the XRD patterns suggested that the etching process did not induce significant changes in the crystalline structure of the glass. The amorphous nature of the glass was maintained throughout the etching treatment regardless of the etching time. These results indicate that the observed changes in the surface morphology and frictional performance are primarily attributed to surface texturing induced by etching rather than structural modifications of the glass. Notably, the absence of any sharp diffraction peaks, even after 60 minutes of etching, indicates that no nanocrystalline phases were formed during the chemical etching process. The etching mechanism involves the dissolution of the silica network rather than phase transformation, preserving the amorphous nature of the glass substrate throughout the treatment.^33,34^

**Fig. 5:**
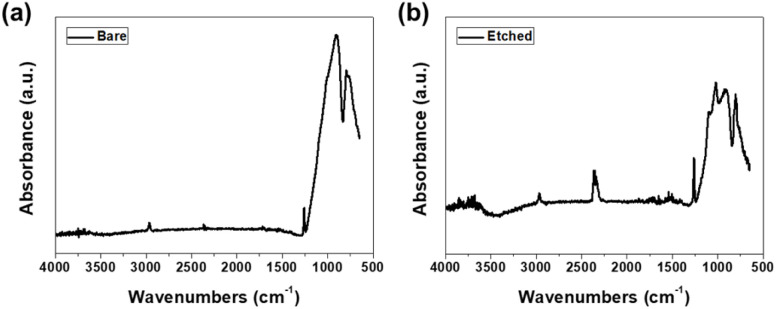
Fourier-transform infrared spectroscopy spectra of (a) bare and (b) etched glass samples.

Fig. 5 shows the FTIR spectra of the bare and etched glass samples. All samples were dried at 60 °C for 2 hours prior to measurement to minimize surface-adsorbed water. The bare glass sample exhibited characteristic peaks at 797, 909, 1260, and 2964 cm^−1^, which can be assigned to various vibrational modes of the silica network. The peak at 797 cm^−1^ corresponds to the bending vibration of the Si–O–Si bond, while the peak at 909 cm^−1^ is attributed to the stretching vibration of the Si–O bond.^35^ The peak at 1260 cm^−1^ was associated with the asymmetric stretching vibration of the Si–O–Si bridges, and the peak at 2964 cm^−1^ was related to the presence of hydroxyl groups (OH) on the glass surface.^36^ In contrast, the etched glass samples showed peaks at 803, 923, 1020, 1097, 1263, and 2967 cm^−1^. Additionally, improved absorption was observed in the 3700 cm^−1^ region for etched samples compared to bare glass. The emergence of these peaks suggests that the etching process induced changes in the chemical composition and functional groups on the glass surface. The peak at 803 cm^−1^ can be attributed to the symmetric stretching vibration of the Si–O–Si bond, while the peak at 923 cm^−1^ is associated with the stretching vibration of Si–OH groups.^37^ The peaks at 1020 and 1097 cm^−1^ are related to the asymmetric stretching vibration of the Si–O–Si bonds, indicating a higher degree of network connectivity in the etched glass samples.^38^ The peak at 1263 cm^−1^ corresponds to the asymmetric stretching vibration of Si–O– Si bridges, similar to that observed in the bare glass sample.^39^ The peak at 2967 cm^−1^ is attributed to the presence of hydroxyl groups on the etched glass surface.^40^ The improved absorption near 3700 cm^−1^ in etched samples corresponds to free silanol groups (Si–OH), which are characteristic of hydroxylated silica surfaces.^41^ The peak at 2350 cm^−1^ observed in both spectra corresponds to atmospheric CO_2_ absorbed during measurement, as FTIR analysis was conducted in ambient conditions.

The observed changes in the FTIR spectra of the etched glass samples can be explained by chemical reactions occurring during the etching process. The etching solution, consisting of deionized water and ammonium bifluoride, selectively removes the silica network, breaks the Si–O bonds, and creates new functional groups on the surface. The increased intensity and broadening of the peaks related to Si–O–Si bonds suggest a higher degree of network connectivity and a more disordered structure in the etched glass samples. This could be due to preferential etching of the less stable regions of the glass network, resulting in a more interconnected and robust surface layer.

The presence of an additional peak related to Si–OH groups, the increased intensity of the hydroxyl peak, and the improved absorption in the 3700 cm^−1^ region in the etched glass samples indicate a significantly higher concentration of hydroxyl groups on the surface. These three distinct spectral changes provide strong evidence for progressive surface hydroxylation with etching treatment. These hydroxyl groups play a crucial role in the formation of a hydrophilic surface layer, which correlates directly with the observed reduction in contact angle and has important implications for the frictional performance of the etched glass.

**Fig. 6:**
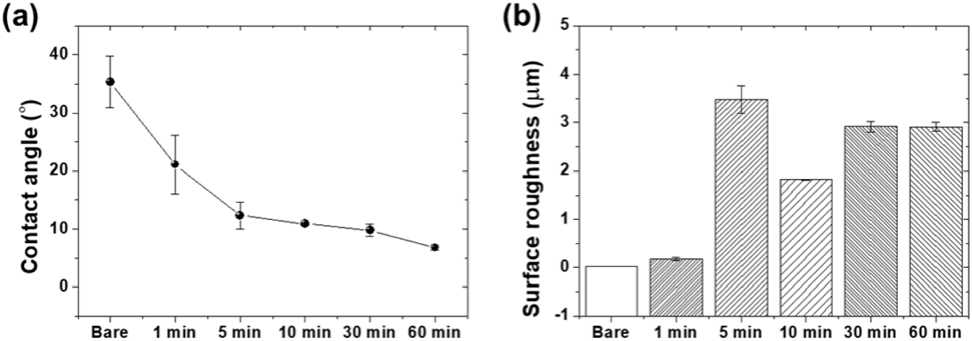
(a) Water contact angle measurements and (b) surface roughness values of glass samples as a function of etching time.

Fig. 6 shows the contact angle measurements and surface roughness values of the glass samples as a function of the etching time. Fig. 6a shows the water contact angle measurements. The bare glass sample exhibited a contact angle of 35.35°, representing the highest contact angle. As the etching time increased, the contact angle gradually decreased, with values of 21.09°, 12.33°, 10.92°, 9.79°, and 6.77° for samples etched for 1, 5, 10, 30, and 60 min, respectively. The significant decrease in the contact angle indicates a substantial improvement in the surface wettability, with the 60-minute etched sample exhibiting the highest hydrophilicity.

The improved wettability can be attributed to increased surface roughness, as shown in Fig. 6b. The surface roughness values of the bare glass and etched samples (1, 5, 10, 30, and 60 min) were 0.02, 0.17, 3.48, 1.82, 2.91, and 2.91 μm, respectively. The surface roughness was the highest at 5 min, followed by a significant decrease after 10 min and a subsequent increase. This suggests that the microstructure was maximized at 5 min and then gradually etched, transitioning to a micro/nanostructure. The higher surface roughness of the etched samples provided a larger surface area and the presence of micro- and nanoscale anisotropy, facilitating the spreading and adhesion of water droplets on the surface.^42^ The decrease in the contact angle with increasing etching time indicates a transition to highly hydrophilic surfaces, which can be beneficial for various applications requiring improved wettability. The increased surface roughness of the etched samples contributed to the improved hydrophilicity, as is evident from the higher *R*_a_ values. The micro- and nanoscale surface features generated by the etching process increase the surface area and provide more sites for water droplets to adhere and spread, leading to lower contact angles. However, it should be noted that the relationship between surface roughness and contact angle is not linear in this study. The 5-minute etched sample, despite having the highest roughness value (3.48 μm), did not exhibit the lowest contact angle. This observation can be attributed to the combined effects of surface morphology and chemical modification. The transition between different wetting states (Wenzel and Cassie–Baxter), along with the progressive increase in surface hydroxyl groups confirmed by FTIR analysis, both contribute to the overall wetting behavior.^43,44^ This demonstrates that surface chemistry plays an equally important role as surface topography in determining the hydrophilicity of etched glass surfaces.

**Fig. 7:**
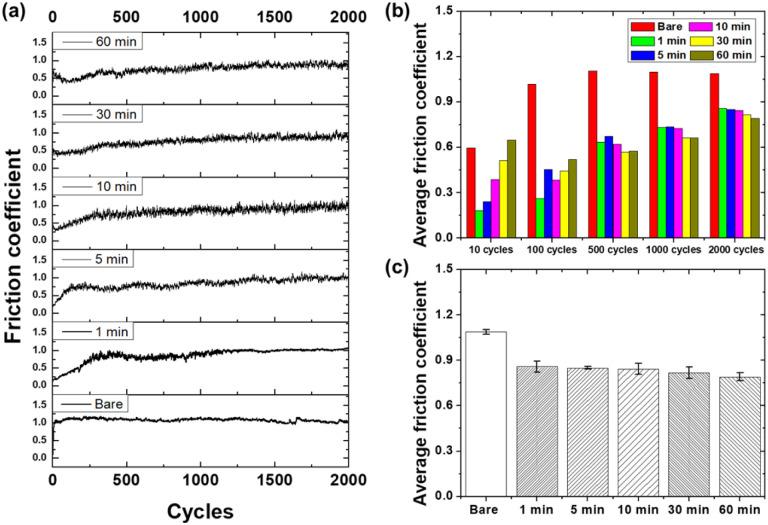
Friction coefficient results for glass samples as a function of etching time: (a) friction coefficient history over 2000 sliding cycles, (b) average friction coefficient at different stages of the sliding test, and (c) overall average friction coefficient.

Fig. 7 presents the friction coefficient results of the glass samples subjected to the sliding tests. The friction coefficient history for 2000 sliding cycles (Fig. 7a) reveals the distinct behaviors of the bare glass and etched samples. The bare glass sample exhibited a rapid increase in the friction coefficient at the beginning of the test, reaching a relatively high value, which then remained stable at approximately 1.08 before slightly increasing after 1700 cycles, ultimately reaching 1.05. The friction coefficient of the bare glass sample remained relatively stable with some fluctuations observed during the initial cycles. This behavior can be attributed to the smooth mating surfaces, allowing direct contact between the surfaces, resulting in high adhesion and friction forces.^45^

In contrast, the etched samples showed a significant reduction in the friction coefficient compared that with of the bare glass. The 1-minute and 5-minute etched samples exhibited lower friction coefficient profiles than the bare sample. The friction coefficient of the 1 minute etched sample gradually increased up to approximately 300 cycles, after which it experienced a slight increase, finally reaching a value of 1.05. In contrast, the 5-minute etched sample showed a rapid increase in the friction coefficient within 100 cycles, reaching approximately 0.75, and then gradually increasing over the remaining 2000 cycles, reaching approximately 1.01 at 2000 cycles. These variations in the friction coefficient can be attributed to surface texturing induced by the etching process, which modifies the contact mechanics and lubrication characteristics at the sliding interface. As the etching time increased, the rate of increase of the friction coefficient of the etched samples further decreased. The average friction coefficient of the 10-minute and 30-minute etched samples gradually increased up to 300 cycles, reaching values of 0.8 and 0.73, respectively, and then maintained relatively constant values. These samples exhibited a more pronounced reduction in the friction coefficient compared to the 1-minute etched sample, indicating a gradual improvement in frictional performance with increasing etching time. The decrease in the friction coefficient can be attributed to the increased surface roughness and the presence of micro- and nanoscale surface features, which reduce the adhesion between the contacting surfaces and facilitate a stable sliding motion.^46^ The 60-minute etched sample showed a decrease to below 0.5 within 100 cycles, and then gradually increased to approximately 0.8 up to 2000 cycles. The friction coefficient profile of the 60-minute etched sample demonstrates a remarkable reduction compared to that of the bare glass and other etched samples. This sample maintained consistently low friction coefficients throughout the sliding test, with minimal fluctuations. The superior frictional performance of the 60-minute etched sample can be attributed to its highly complex surface morphology, with densely networked micro- and nanoscale features. The friction reduction mechanism involves both structural and chemical contributions. The surface texture provides micro-reservoirs for debris entrapment, while the hydroxylated surface enhances the formation of a boundary lubrication layer through hydrogen bonding with ambient moisture.

Fig. 7b shows the average friction coefficients at various stages (10, 100, 500, 1000, and 2000 cycles) of the sliding test for all the samples. The bare glass sample exhibited the highest average friction coefficients in the stages after 100 cycles, with values of 0.59, 1.02, 1.10, 1.10, and 1.09 for 10, 100, 500, 1000, and 2000 cycles, respectively. In contrast, the etched samples exhibited lower average friction coefficients in most of the stages. The average friction coefficients for the 60-minute etched sample were 0.65, 0.52, 0.57, 0.66, and 0.79 for 10, 100, 500, 1000, and 2000 cycles, respectively. These results indicate that the surface texturing induced by chemical etching effectively reduced friction, and this reduction was sustained throughout the sliding test.

Fig. 7c presents the overall average friction coefficients for all the samples. The bare glass sample had the highest overall average friction coefficient of 1.09, whereas the etched samples exhibited gradually lower values with increasing etching time. The 60-minute etched sample had the lowest overall average friction coefficient of 0.79 which represents an approximately 27% reduction compared to bare glass. One of the primary mechanisms responsible for friction reduction in etched glass samples is the entrapment of wear debris and the formation of tribofilms at the sliding interface.^47^ During the sliding process, wear particles are generated owing to the abrasive interactions between the mating surfaces. In the case of the bare glass sample, these wear particles could easily accumulate at the interface, leading to third-body wear and increased friction. However, the surface features present on the etched glass samples act as micro-reservoirs, effectively trapping wear debris and preventing their continuous exposure to the abrasive process. The capture of wear particles helps to reduce the abrasive component of friction and maintain a smoother sliding interface. Furthermore, the trapped wear debris, along with the increased surface roughness and presence of hydroxyl groups on the etched glass surfaces, can promote the formation of protective tribofilms. Tribofilms are thin, mechanically, and chemically altered layers that form at the sliding interface owing to the complex interactions between mating surfaces, wear debris, and the environment.^48^ Tribofilms can consist of a mixture of oxidized wear particles, fragmented material, water molecules, and adsorbed species from the environment, such as hydroxyl groups. When a stable and lubricious tribofilm is formed, it significantly reduces the direct contact between mating surfaces, leading to reduced friction and wear. The surface texture of the etched glass samples also influences the contact mechanics and stress distribution at the sliding interface. The micro- and nanoscale surface features induce a localized elastic deformation of the contacting asperities, promoting a more compliant contact.^49^ The increased surface compliance allows for a more uniform distribution of contact stresses, reducing the maximum stresses and likelihood of plastic deformation or fracture. The reduction in contact stresses and more uniform stress distribution contributed to the lower friction and improved wear resistance of the etched glass samples. In addition, the surface texture can influence the lubrication regime at the sliding interface. The presence of microreservoirs on etched glass surfaces can easily retain lubricants, such as water molecules or adsorbed species from the environment. These lubricants can form a thin protective film that separates the mating surfaces and reduces direct solid–solid contact. The lubrication effect was improved by the increased surface hydrophilicity of the etched glass samples, which was attributed to the lower contact angles. The hydrophilic nature of the etched surfaces promoted the spreading and adhesion of the lubricating film, providing a more stable and effective low-friction performance. Overall, the friction test results demonstrated the potential of chemical etching as a surface-modification technique for improving the frictional performance of glass surfaces. By controlling the etching time, the surface roughness and morphology could be tailored to achieve the desired friction and wear characteristics. The optimized surface texture obtained through chemical etching can significantly reduce friction, improve lubricity, and improve wear resistance, making it a promising approach for various applications in which improved frictional performance is desired.

**Fig. 8:**
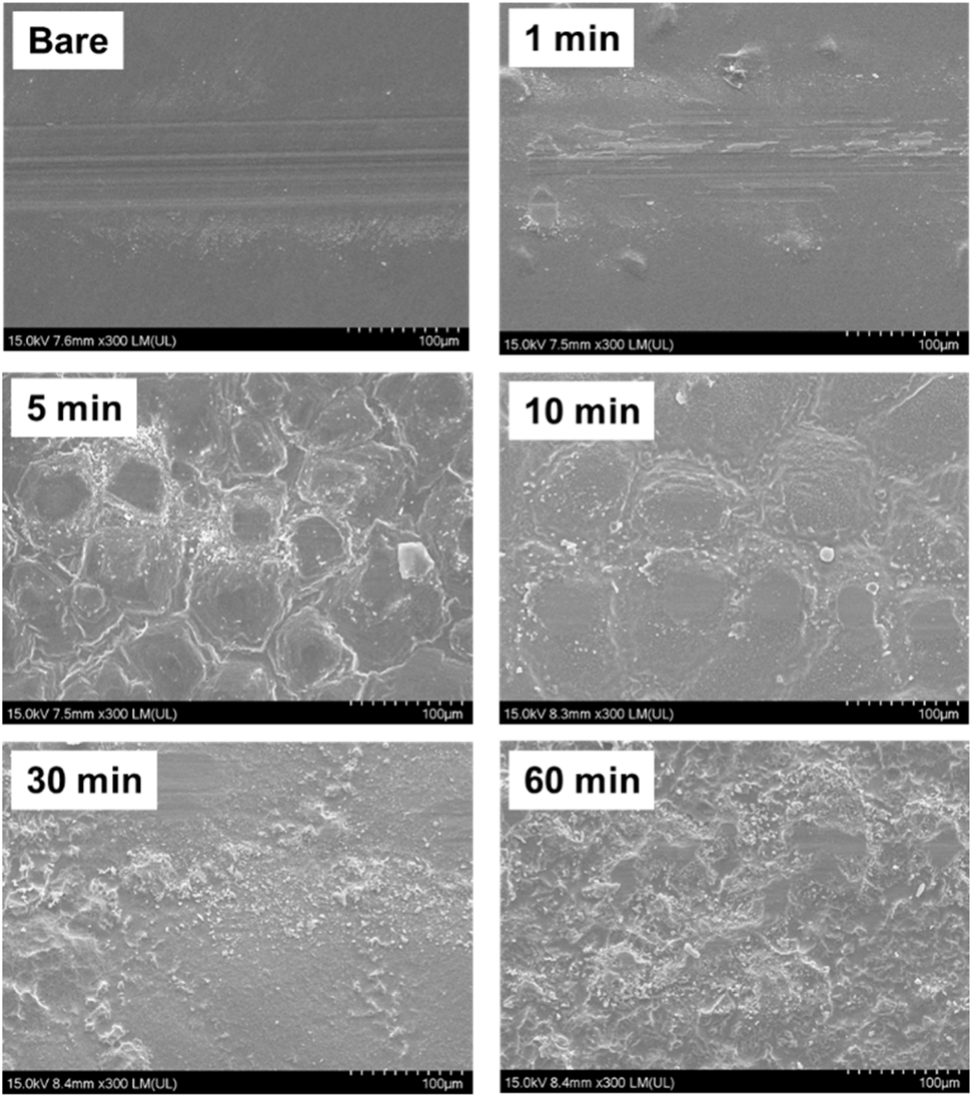
Scanning electron microscopy images of wear tracks formed on glass samples after sliding tests.

Fig. 8 shows the SEM images of the wear tracks formed on the glass samples after the sliding tests. The bare glass sample exhibited a wide and deep wear track, indicating severe material removal and surface damage during the sliding tests. The wear track of the bare glass sample was characterized by substantial plowing and scratching, suggesting a high level of abrasive wear. The presence of large wear debris particles and the rough appearance of the wear track further confirmed the poor wear resistance of bare glass.

In contrast, the etched samples showed progressively narrower and shallower wear tracks with increasing etching times. The 1-minute etched sample exhibited a slight reduction in the width and depth of the wear track compared to bare glass, indicating an improvement in wear resistance. The wear track of the 1-minute etched sample appeared smoother and less severe, with fewer signs of plowing and scratching. As the etching time increased to 5, 10, and 30 min, the wear tracks became increasingly narrower and shallower, indicating further improvement in the wear resistance. The reduction in wear track dimensions can be attributed to the surface texturing induced by the etching process, which modifies the contact mechanics and distributes the contact pressure, thereby mitigating surface damage. The etched samples with etching times of 10 min and above exhibited minimal wear with barely visible wear tracks. The wear tracks of these samples were significantly narrower and shallower than those of the other samples, indicating their excellent wear resistance. This can be attributed to the dense network of micro- and nanoscale features on the etched samples, which effectively reduced the direct contact between the mating surfaces and distributed the contact stresses more evenly. This surface morphology helps minimize abrasive wear and surface damage, resulting in minimal material loss and the formation of barely noticeable wear tracks. The gradual decrease in the wear track dimensions with increasing etching time demonstrates the progressive improvement in wear resistance achieved through chemical etching.

To quantitatively assess wear behavior, surface roughness measurements were performed within the wear tracks after 2000 sliding cycles. The bare glass showed moderate smoothening from *R*_a_ = 0.02 μm to 0.012 μm (40% reduction), indicating polishing-type wear typical of hard, brittle materials. The 1-minute etched sample exhibited minimal change (0.17 to 0.156 μm, 8.2% reduction), suggesting that even short etching times provide wear protection.

Remarkably, the 10-minute etched sample showed near-perfect roughness preservation, with only 0.8% reduction (1.82 to 1.805 μm). This exceptional roughness retention explains its superior tribological performance throughout the sliding tests. The 5-minute sample, despite having the highest initial roughness (3.48 μm), maintained substantial texture after wear (2.126 μm), though with 38.9% reduction due to asperity deformation.

The 30 and 60-minute samples showed intermediate behavior with approximately 30–34% roughness reduction, maintaining sufficient texture (1.933 and 2.052 μm respectively) to sustain low friction conditions.

The 10-minute etched sample showed remarkable roughness preservation, maintaining *R*_a_ of 1.805 μm within the wear track. This minimal change indicates excellent wear resistance through optimal surface texture that effectively distributes contact stresses and accommodates wear debris.

In contrast, the 5-minute sample with the highest initial roughness (*R*_a_ = 3.48 μm) experienced 38.9% reduction to 2.126 μm, suggesting partial asperity flattening under load. However, it still maintained higher absolute roughness than most other samples, contributing to sustained friction reduction.

The 1-minute sample showed minimal wear with only 8.2% reduction, demonstrating that even short etching times provide some wear protection through surface chemistry modification.

Surface texturing modifies the contact geometry, disperses the local contact pressures, and enhances the load-bearing capacity of glass surfaces. Additionally, surface texturing promotes the formation of stable lubricating films, which helps separate the mating surfaces and reduce direct contact. The presence of lubricating films contributes to the reduction in adhesive wear and the decrease in shear stress at the sliding interface, improving the wear resistance of the etched samples. The decreased severity of the wear tracks, smoothening of the surface, and reduction in plowing and scratching marks on the etched samples confirmed the effectiveness of chemical etching in mitigating surface damage and enhancing wear performance. In other words, the application of micro/nanostructures through etching highlights the potential of surface texturing to improve the wear resistance of glass surfaces. By optimizing the etching time and surface morphology, significant improvements in wear resistance can be achieved, thereby extending the lifetime and reliability of glass components in tribological applications. Wear track analysis complements the friction coefficient results, demonstrating the superior friction and wear performance of the etched glass samples.

**Fig. 9:**
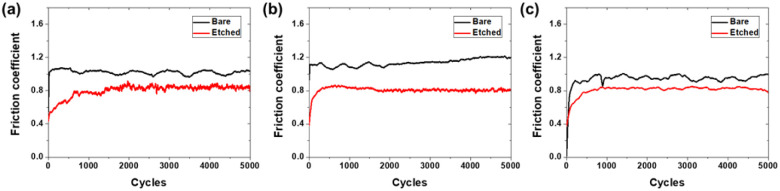
Friction coefficient results for bare and etched glass (10 min) under different normal loads: (a) 20 mN, (b) 30 mN, and (c) 50 mN.

Fig. 9 presents the friction coefficient results of the bare glass and 10-minute etched samples at various loads (20, 30, and 50 mN) during the sliding tests. At a load of 20 mN (Fig. 9a), the bare glass sample exhibited a relatively high friction coefficient throughout the sliding test with an average value of 1.02. The friction coefficient profile of the bare glass sample showed some fluctuations but remained relatively stable over 5000 cycles. In contrast, the 10-minute etched sample displayed a lower friction coefficient profile, with an average value of 0.80. The friction coefficient of the 10-minute etched sample was consistently lower than that of the bare glass sample throughout the sliding tests. This reduction in the friction coefficient can be attributed to the improved stress distribution effects and reduced direct contact between the mating surfaces owing to surface texturing induced by the etching process. Similar trends were observed under the load conditions of 30 mN (Fig. 9b) and 50 mN (Fig. 9c). The bare glass sample exhibited higher friction coefficients than the 10-minute etched sample under both load conditions. The average friction coefficients of the bare glass samples were 1.14 and 0.94 at normal loads of 30 and 50 mN, respectively. In contrast, the 10-minute etched sample maintained a lower average friction coefficients of 0.81 in both load conditions. The consistent friction coefficient values of the 10-minute etched sample across various loads highlight the robustness and effectiveness of surface texturing in reducing friction even at increased loads. The friction coefficient results demonstrate the superior frictional performance of the etched glass sample compared that with of the bare glass sample under various loads. Surface texturing induced by chemical etching has proven to be effective in reducing friction regardless of the applied load. The micro- and nanoscale surface features generated by the etching process can improve the frictional characteristics, promote the formation of stable lubricating films, and reduce the direct contact between the mating surfaces, leading to lower friction coefficients. The consistent reduction in the friction coefficient observed for the 10-minute etched sample across different normal loads suggests that surface texturing maintains its effectiveness even under increased loading conditions. This finding is significant because it highlights the potential of chemical etching as a surface modification technique for improving the frictional performance of glass surfaces in various applications where different loads or localized stresses may be encountered.

**Fig. 10:**
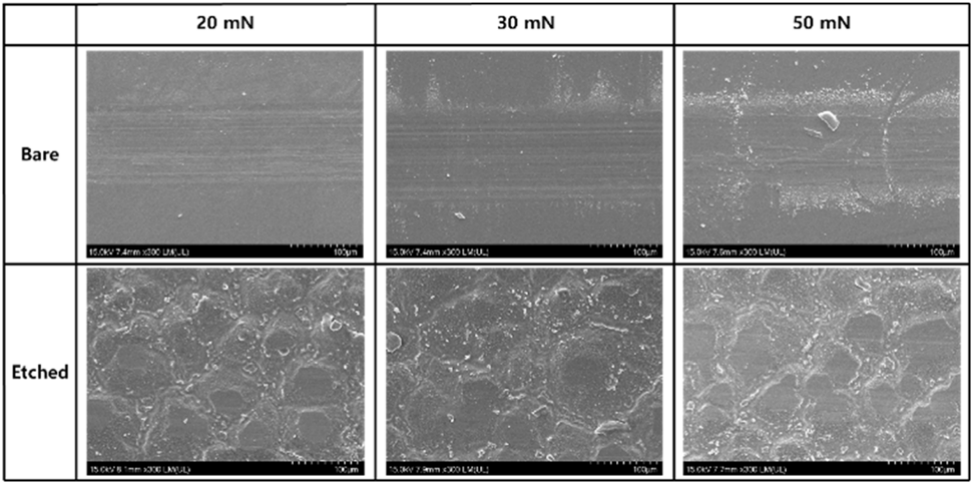
Scanning electron microscope images of the wear tracks formed on bare and etched glass (10 min) under different normal loads.

Fig. 10 shows SEM images of the wear tracks formed on the bare glass and 10-minute etched samples at various loads (20, 30, and 50 mN). At a load of 20 mN, the bare glass sample exhibited a relatively wide and deep wear track, indicating a substantial material removal and surface damage. The wear track of the bare glass sample showed signs of plowing and scratching, suggesting an abrasive wear mechanism. In contrast, the wear track of the 10-minute etched sample appeared narrower and shallower, indicating an improved wear resistance. Surface texturing induced by the etching process can disperse local contact pressures and improve the load-bearing capacity of the glass surface, thereby reducing surface damage. Similar observations were made for the wear tracks under loads of 30 and 50 mN. The bare glass sample consistently showed wider and deeper wear tracks compared to the 10-minute etched sample. As the load increased, the wear tracks of both the samples became more pronounced with increasing width and depth. However, the 10-minute etched sample maintained its superiority in terms of wear resistance, exhibiting narrower and shallower wear tracks under all the loading conditions. Wear track analysis complements the friction coefficient results, confirming the improved wear resistance of the etched glass sample compared to that of the bare glass sample. Surface texturing induced by chemical etching contributes to wear reduction by modifying contact mechanics, dispersing local contact pressures, and promoting the formation of stable lubricating films. Additionally, the entrapment of wear debris within the surface features helps minimize third-body wear and maintain a smoother sliding interface. The decreased severity of the wear tracks, characterized by narrower and shallower profiles, in the 10-minute etched sample confirms the effectiveness of chemical etching in mitigating surface damage and improving wear performance under various loads.

**Fig. 11:**
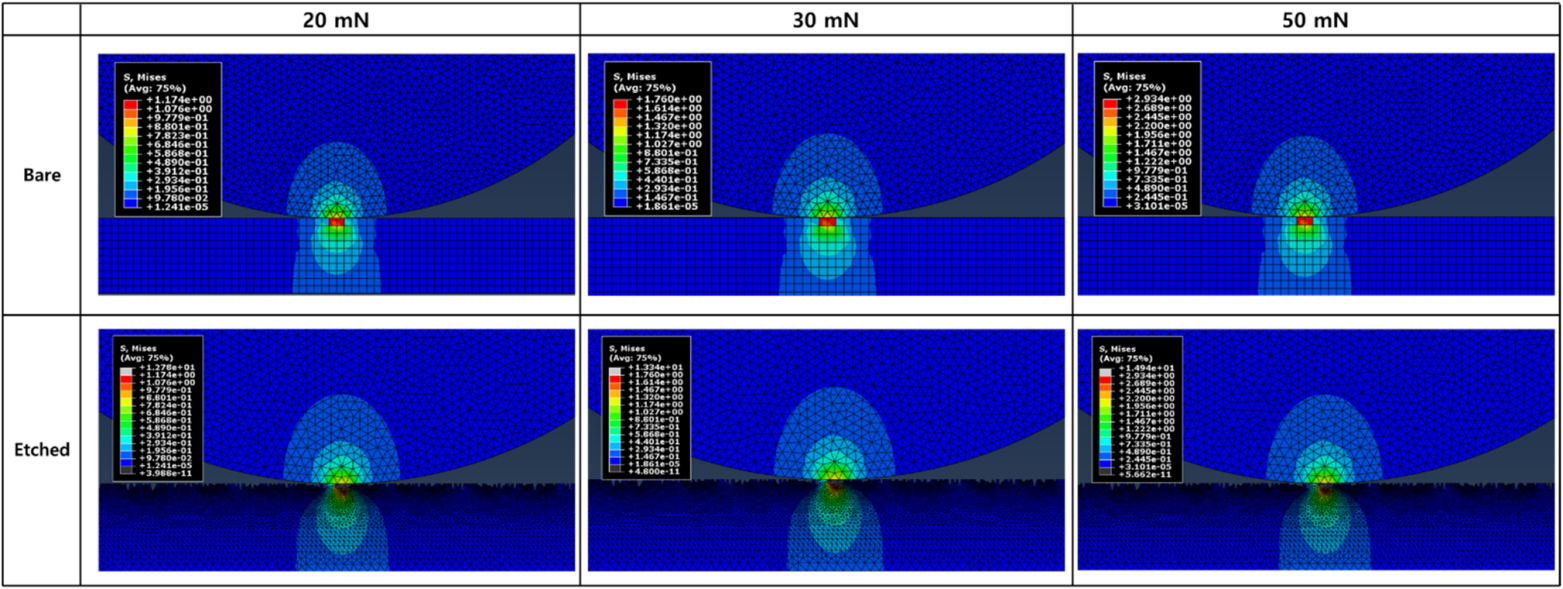
Finite element analysis (FEA) simulation results of stress distribution in bare glass and etched glass during indentation under different normal loads.

Fig. 11 shows the finite element analysis (FEA) simulation results of the stress distribution for the bare glass and etched glass samples during indentation at different normal loads (20, 30, and 50 mN). The etched glass sample exhibited higher maximum stress values than those of the bare glass sample under the same loading conditions. These observations can be attributed to surface irregularities and protrusions induced by the chemical etching process, which can lead to stress amplification. The higher stress values in the etched glass samples can be explained by the non-uniform surface topography resulting from the etching treatment. Micro- and nanoscale surface features, such as peaks and valleys, act as stress raisers, promoting stress localization and intensification at the contact interface. When the etched glass surface was in contact, the applied load was initially borne by the protruding asperities, resulting in a smaller effective contact area compared to the smooth surface of the bare glass sample. Consequently, the contact pressures and stress levels were significantly higher at these asperity contacts, leading to an observed increase in the maximum stress values. However, despite the higher peak stresses, the etched glass sample showed a reduced range of stress concentration compared with the bare glass sample. These results can be reconciled by considering the beneficial effects of surface texturing on the overall stress distribution and contact mechanics, as evidenced by the superior tribological performance in terms of friction and wear reduction, as observed in the friction coefficient results and wear track analysis. Surface texturing induced by chemical etching plays a crucial role in redistributing contact stresses and enhancing the load-bearing capacity of glass surfaces. The presence of micro- and nanoscale surface features increases the effective contact area, allowing a more uniform stress distribution across the textured surface.^50^ As the applied load is shared among more asperities, the individual asperity contacts experience lower contact pressures, resulting in a reduction in the overall stress levels in the bulk material. Furthermore, surface texturing helps reduce direct asperity contact, mitigate wear, and improve the low-friction performance of the sliding interface. The entrapped wear particles and lubricants act as protective tribofilms, preventing direct contact between the mating surfaces and further reducing friction and wear.

**Fig. 12:**
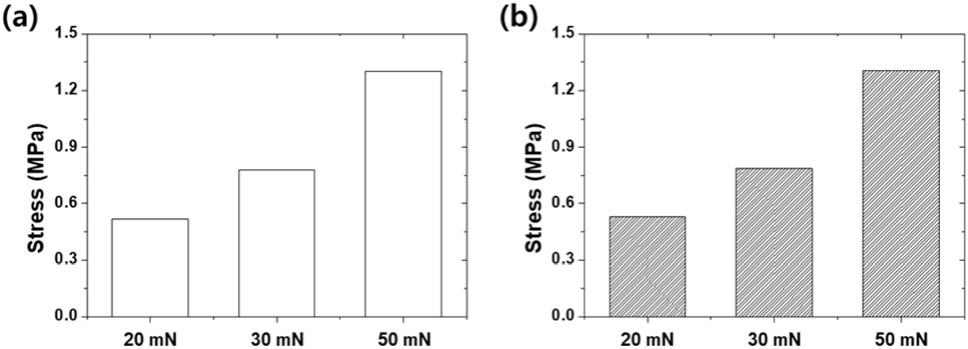
Stress behavior analysis of bare and etched glass during indentation comparing the maximum stresses on the counter tip: (a) bare glass and (b) etched glass.

Fig. 12 shows the maximum stresses on the counter tip for both the bare glass sample and etched glass sample under different normal loads (20, 30, and 50 mN). For the bare glass sample, the maximum stresses on the substrate were 0.52, 0.78, and 1.30 MPa for loads of 20, 30, and 50 mN, respectively. In contrast, the etched glass sample exhibited slightly higher maximum stresses on the substrate compared to the bare glass sample, with values of 0.53, 0.79, and 1.30 MPa for the corresponding loads. The ability of the etched glass sample to maintain stable stress responses on the substrate despite the increase in load can be attributed to the beneficial effects of surface texturing. The micro- and nanoscale surface features of the etched glass sample helped distribute the contact stresses more uniformly across the surface, preventing excessive stress accumulation on the substrate. The quantitative stress values further supported the overall analysis and conclusions derived from the FEA simulation results. Surface texturing induced by chemical etching not only reduces the stresses experienced by the substrate surface under contact with the counter tip but also promotes a more stable stress response on the substrate, even when the load increases. These findings emphasize the importance of considering both the counter tip and substrate stresses when evaluating the tribological performance of textured materials. The ability of the etched glass sample to maintain a stable stress behavior demonstrates its superior frictional characteristics compared to the bare glass sample. These results contribute to a deeper understanding of the underlying mechanisms of the improved tribological performance of textured glass and can aid in the optimization of the surface texturing parameters for specific applications.

## 4. Conclusions

The extensive analysis of the experimental results and FEA simulations in this study shed light on the effect of surface texturing induced by chemical etching on the frictional behavior and wear resistance of glass surfaces. The chemical etching process effectively modified the surface morphology, creating micro- and nanoscale features that substantially improved the tribological performance of glass. The surface characterization revealed that chemical etching altered the surface topography, surface roughness, and wettability. Reciprocating sliding tests validated the superior frictional characteristics of the etched glass samples, with reduced friction coefficients and less severe wear tracks compared to bare glass. FEA simulations revealed the stress distribution and contact mechanics, emphasizing the advantageous effects of surface texturing in dispersing contact stresses and promoting stable stress responses. The results suggest that the improved tribological performance may be related to several mechanisms, including efficient dispersion of contact stresses, formation of stable lubricating films, reduction of direct asperity contact, and entrapment of wear debris within the surface features. The outcomes of this study contribute to the progress of surface engineering and provide a foundation for the design and optimization of surface texturing to improve friction and wear performance in various applications. The use of chemical etching as a surface modification technique presents immense potential for industries in which glass components are subjected to frictional contact. Further investigations considering the influence of the operating conditions and counter surface materials can offer a better understanding of the tribological behavior of etched glass surfaces. This study highlights the impact of surface texturing induced by chemical etching on the frictional behavior and wear resistance of glass surfaces, paving the way for the development of high-performance glass components with enhanced tribological characteristics. Future investigations must address the critical need for extended durability testing, as industrial applications typically require tribological performance over sliding distances on the order of kilometers—approximately 1000 times longer than cumulative distance tested in this study. While the current results demonstrate promising friction reduction during initial sliding stages, long-term testing over industrially relevant distances following standards such as JIS R 1613-1993 is essential to validate the persistence of these improvements. Additionally, investigations considering different operating conditions and counter surface materials, along with surface treatment optimization to maintain the hydroxyl-rich surfaces over extended periods, will be crucial for practical industrial implementation.

## Author contributions

Sung-Jun Lee: conceptualization, methodology, software, validation, formal analysis, investigation, data curation, writing – original draft, writing – review & editing, visualization. Chang-Lae Kim: conceptualization, methodology, resources, writing – review & editing, supervision, project administration.

## Conflicts of interest

There are no conflicts to declare.

## Data Availability

All data supporting this study are included in the article.

## References

[cit1] Chen X., Yu N., Song Y., Liu T., Xu H., Guan D., Li Z., Huang W. H., Shao Z., Ciucci F. (2024). Adv. Mater..

[cit2] Mohanty S., Basak S., Saran D., Chatterjee K., Datta T., Kumar A., Prakash C., Chun D.-M., Hong S.-T., Sahu K. K. (2024). Int. J. Precis. Eng. Manuf..

[cit3] Ramezani M., Mohd Ripin Z., Pasang T., Jiang C.-P. (2023). Metals.

[cit4] Wang P., Liang H., Jiang L., Qian L. (2023). Wear.

[cit5] Şirin Ş., Akıncıoğlu S., Gupta M. K., Kıvak T., Khanna N. (2023). Tribol. Int..

[cit6] Yao J., Wu Y., Sun J., Tian J., Zhou P., Bao Z., Xia Z., Gao L. (2021). Mater. Res. Express.

[cit7] Binali R., Demirpolat H., Kuntoğlu M., Sağlam H. (2023). Lubricants.

[cit8] Makhesana M. A., Patel K. M., Krolczyk G. M., Danish M., Singla A. K., Khanna N. (2023). CIRP J. Manuf. Sci. Technol..

[cit9] Tabrizi A., Aghajani H., Laleh F. (2023). Exp. Tech..

[cit10] Korkmaz M. E., Gupta M. K., Günay M., Boy M., Yaşar N., Demirsöz R., Ross K. N. S., Abbas Y. (2023). J. Manuf. Process..

[cit11] Ralls A. M., Leong K., Liu S., Wang X., Jiang Y., Menezes P. L. (2024). Wear.

[cit12] Fujita N., Yamaguchi H., Kinoshita T., Iwao M., Nakanishi Y. (2022). Tribol. Int..

[cit13] He H., Qiao Q., Xiao T., Yu J., Kim S. H. (2022). J. Am. Ceram. Soc..

[cit14] Jayarama A., Kannarpady G. K., Kale S., Prabhu S., Pinto R. (2022). Mater. Today: Proc..

[cit15] Butkutė A., Baravykas T., Stančikas J., Tičkūnas T., Vargalis R., Paipulas D., Sirutkaitis V., Jonušauskas L. (2021). Opt. Express.

[cit16] Kargozar S., Mozafari M., Ghodrat S., Fiume E., Baino F. (2021). Mater. Sci. Eng., C.

[cit17] Gavinho S., Graça M., Prezas P., Kumar J. S., Melo B., Sales A., Almeida A., Valente M. (2021). J. Non-Cryst. Solids.

[cit18] Badán J. A., Navarrete-Astorga E., Henríquez R., Jiménez F. M., Ariosa D., Ramos-Barrado J. R., Dalchiele E. A. (2022). Nanomaterials.

[cit19] Ramasamy N., Abul Kalam M., Varman M., Teoh Y. H. (2021). Coatings.

[cit20] Bilaç O., Duran C. (2023). Int. J. Appl. Ceram. Technol..

[cit21] Maximov M., Maximov O.-C., Craciun L., Ficai D., Ficai A., Andronescu E. (2021). Coatings.

[cit22] Saha S., Roy S. (2022). Materials.

[cit23] Lee S.-J., Kim G.-M., Kim C.-L. (2023). Polym. Test..

[cit24] Lee S.-J., Segu D. Z., Kim C.-L. (2024). Phys. Scr..

[cit25] Lee S.-J., Kim C.-L. (2024). Soft Matter.

[cit26] Judge J. S. (1971). J. Electrochem. Soc..

[cit27] Knotter D. M. (2000). J. Am. Chem. Soc..

[cit28] Lim Y., Ha S. (2023). SoftwareX.

[cit29] Lee S.-J., Kim C.-L. (2023). RSC Adv..

[cit30] Lee S.-J., Kim G.-M., Kim C.-L. (2023). RSC Adv..

[cit31] Konstantinova T., Andronic M., Baklykov D., Stukalova V., Ezenkova D., Zikiy E., Bashinova M., Solovev A., Lotkov E., Ryzhikov I. (2023). Sci. Rep..

[cit32] Chakraborty R., Dey A., Mukhopadhyay A. K. (2010). Metall. Mater. Trans..

[cit33] Konstantinova T. G., Andronic M. M., Baklykov D. A., Stukalova V. E., Ezenkova D. A., Zikiy E. V., Bashinova M. V., Solovev A. A., Lotkov E. S., Ryzhikov I. A., Rodionov I. A. (2023). Sci. Rep..

[cit34] Spierings G. A. C. M. (1993). J. Mater. Sci..

[cit35] Ibrahim M. M., Fanny M. A., Hassaan M., ElBatal H. (2016). Silicon.

[cit36] Link M. M., Lin Y.-T., Banerjee J., Smith N. J., Ogrinc A. L., Guo Y., Li Y.-S., Yoo S., Oh K., Choi J. (2025). Langmuir.

[cit37] Dinh T.-H., Ngo C.-V., Chun D.-M. (2020). Appl. Phys. A.

[cit38] Rincón A., Desideri D., Bernardo E. (2018). J. Cleaner Prod..

[cit39] Gautam C., Yadav A. K., Singh A. K. (2012). Int. Scholarly Res. Not..

[cit40] Abdullah M., Yahya N., Kasim A., Saipuddin S. F. (2018). Int. J. Eng. Technol..

[cit41] Efimov A. M., Pogareva V. G., Shashkin A. V. (2003). J. Non-Cryst. Solids.

[cit42] Lee S.-J., Segu D. Z., Kim C.-L. (2024). RSC Adv..

[cit43] Kumar P., Harvie D. J. E. (2024). Langmuir.

[cit44] Myronyuk O., Vanagas E., Rodin A. M., Wesolowski M. (2024). Materials.

[cit45] Lee S.-J., Kim C.-L. (2024). Polym. Test..

[cit46] Ryu B.-H., Kim D.-E. (2015). CIRP Ann..

[cit47] Gosvami N. N., Bares J., Mangolini F., Konicek A., Yablon D., Carpick R. (2015). Science.

[cit48] Guo L., Pei X., Zhao F., Zhang L., Li G., Zhang G. (2020). Tribol. Int..

[cit49] Kim G.-M., Lee J.-W., Lee S.-J., Kim C.-L. (2022). Materials.

[cit50] Lee S.-J., Kim G.-M., Kim C.-L. (2021). Coatings.

